# Journal statistics, Scopus pre-evaluation, and appreciation for 2019 reviewers of the *Korean Journal of Women Health Nursing*

**DOI:** 10.4069/kjwhn.2020.03.16

**Published:** 2020-03-18

**Authors:** Sue Kim

**Affiliations:** College of Nursing, Yonsei University, Seoul, Korea

It is both an honor and hefty responsibility to take on the position of editor-in-chief for the Korean Journal of Women Health Nursing (KJWHN) for the period of January 2020 to December 2021. As our editorial board and I attempt to follow the footsteps of our predecessors who have dedicated themselves to the journal’s development, we thank our readers and the authors who have helped KJWHN reach its 25th anniversary this year since the first issue was published in 1995.

In this editorial, I hope to share with readers some information on KJWHN’s journal statistics, preparations for seeking Scopus status, and the necessary directions that follow. I also wish to note appreciation for our 2019 reviewers.

## Journal statistics and citation information of KJWHN

The data on manuscripts submitted to this journal up to December 24, 2019, is presented in Table 1.

The total cites in Scopus as of March 5, 2020, shows 229 documents from KJWHN cited 607 times (data as secondary documents). Although KJWHN is not yet Scopus status, it is certainly being utilized and cited by the international audience, and we intend to expand and increase this trend.

As for the Scopus CiteScore, which is calculated from the previous three years of publications [1], KJWHN’s CiteScore for 2019 can be estimated as 0.148, as follows:

( REF ( "Korean Journal of Women Health Nursing" 2016 ) OR REF ( "Korean J Women Health Nurs" 2016 ) ) AND PUBYEAR = 2019 12

( REF ( "Korean Journal of Women Health Nursing" 2017 ) OR REF ( "Korean J Women Health Nurs" 2017 ) ) AND PUBYEAR = 2019 2

( REF ( " Korean Journal of Women Health Nursing " 2018 ) OR REF ( "Korean J Women Health Nurs" 2018 ) ) AND PUBYEAR = 2019 2

These cites relating to the period of 2019 (12+2+2=16) were divided by the number of articles in 2016–2018 (108). Thus the 2019 CiteScore was 0.148 (16/108). This means the ranking of KJWHN is at about 15% level in general nursing category.

## Scopus pre-evaluation

Among the 16 items listed in the pre-evaluation for Scopus submission system [2], currently our journal status shows complete readiness. The editorial board continues to seek ways to ensure better infrastructure and expand the diversity of potential authors, while making KJWHN a better platform for quality manuscripts to be shared. The Principles for Transparency and Best Practices statement, newly added to the journal website, is an example of our preparation for seeking Scopus status, as well as for registration in other international databases in the near future.

## Future directions for KJWHN

Please note that the journal homepage has been renewed with new functions. The section titled “*Issues and Perspectives*” has been re-named from its prior form ‘*Current Issues*’ to include a wider spectrum of expert opinions and issues of relevance for our readers. We hope this peer-reviewed piece will engage readers to discuss further and perhaps respond via the journal. We are also starting a new section titled “*In this Issue*,” to brief readers to be able to quickly identify the content of each journal issue.

Also, from the March 2020 issue, the following are provided as new functions of the journal homepage: First, the text can be read easily with 80 languages in the world through Google Translate by selecting the preferred language on our homepage.

Second, the author index is provided for better search of specific author’s work.

Third, a graph of journal metrics is presented based on Crossref metadata. Therefore, hit and download count, resolution report of the prefix of the journal, yearly Crossref citation, and source titles of citing articles are easily verified.

Finally, the citation reports based on Crossref Metadata, Scopus, and the Web of Science Core Collection are provided, including citing journals that deposit digital object identifier (DOI) of each article and its references to Crossref.

I believe the above functions will clarify the present position of the journal in the international scholarly journal network and hope KJWHN will be more accessible to readers across the world.

The editorial board of KJWHN is also actively considering various ways to make the journal more attractive and relevant to our readers and potential authors. Our new editorial board includes Dr. Emily Drake, former president of the Association of Women’s Health, Obstetrics, and Neonatal Nursing (AWHONN) [3], and Dr. Yoko Shimpuku, an internationally renowned expert in midwifery [4].

In addition to original articles, we welcome quality review papers and method papers that fit the aims and scope of our journal. Please note the updated author guidelines and statement of research and publication ethics that will be applied from the June 2020 issue, which are available online.

## Appreciation for 2019 reviewers

Last but not least, gratitude goes to the following reviewers who assisted KJWHN in 2019. Without your sharp eyes and suggestions for improvement, publications would not have been possible.

Ahn, Suk Hee (Chungnam National University)

Chae, Hyun Ju (Joongbu University)

Cho, Ok Hee (Kongju National University)

Choi, So Young (Gyeongsang National University)

Chun, Na Mi (Sungshin Women’s University)

Chung, Chae Weon (Seoul National University)

Han, Yong Hee (Hallym Polytechnic University)

Hong, Se Hoon (CHA University)

Hur, Myung Haeng (Eulji University)

Hwang, Kyung Hye (Suwon Science College)

Hwang, Moon Sook (Woosuk University)

Jeong, Geum Hee (Hallym University)

Jun, Eun Mi (Pai Chai University)

Jun, Eun Young (Daejeon University)

Kang, Sook Jung (Ewha Womans University)

Kim, Hee Sook (Dongnam Health University)

Kim, Hyun Kyoung (Kongju National University)

Kim, Kyung Won (Daegu Hanny University)

Kim, Mi Jong (Hannam University)

Kim, Moon Jeong (Pukyung National University)

Kim, Myoung hee (Semyung University)

Kim, Sun-Hee (Daegu Catholic University)

Kim, Young Hee (Dongguk University)

Kim, Young Ju (Daejeon Health Institute of Technology)

Kim, Yun Mi (Eulji University)

Kim, Yun Mi (Gachon University)

Lee, Soo Yeon (Koje University)

Lee, Yun Jung (Seoul Women’s College of Nursing)

Moon, So Hyun (Chosun University)

Park, Hye Sook (Dongyang University)

Park, Hyun Jung (Pyeongtaek University)

Park, Mi Kyung (Nambu University)

Park, So Mi (Yonsei University)

Shin, Gi Soo (Chung-Ang University)

Sung, Mi Hae (Inje University)

Yeo, Jung Hee (Dong-A University)

Yoon, Ji Won (Shinhan University)

As 2020 has been designated as the “Year of the Nurse and Midwife” by the World Health Organization [5] to mark the bicentenary of Florence Nightingale’s birth, our journal will celebrate this memorable year through the continuous publication of cutting edge results for women’s health nursing.

KJWHN relies on the hard work on the part of the editorial team and our committed reviewers, but more importantly, the support of members of the Korean Society of Women Health Nursing and our readers-at-large. We look forward to your input to make 2020 an exciting year of scholarly activities and exchange.

## Figures and Tables

**Table 1.** Basic statistics on manuscripts submitted to the Korean Journal of Women Health Nursing in 2019

Category	Data	Notes
Manuscripts submitted (n)	39	
Manuscripts determined without review (n)	3	Not in accord with journal’s aims and scope
Manuscripts reviewed and determined (n)	35	Accepted and published 30, rejected 5
Manuscripts under review or revision (n)	1	Under review
Acceptance rate (%)	78.9	30/38=0.789
Median time from submission to publication (days)	36	
